# Incidence, risk, and associated factors of depression in adults with physical and sensory disabilities: A nationwide population-based study

**DOI:** 10.1371/journal.pone.0175141

**Published:** 2017-03-31

**Authors:** Szu-Ching Shen, Kuang-Hua Huang, Pei-Tseng Kung, Li-Ting Chiu, Wen-Chen Tsai

**Affiliations:** 1 Department of Public Health, China Medical University, Taichung, Taiwan, R.O.C; 2 Department of Health Services Administration, China Medical University, Taichung, Taiwan, R.O.C; 3 Department of Strategy Planning, Buddhist Dalin Tzu Chi Hospital, Chiayi, Taiwan, R.O.C; 4 Department of Healthcare Administration, Asia University, Taichung, Taiwan, R.O.C; University of Rochester, UNITED STATES

## Abstract

**Background:**

Physical disability has been associated with the risk of depression. We examined the incidence, risk, and associated factors of depression in Taiwanese adults with physical/sensory disabilities.

**Methods:**

Two national databases were used to retrospectively analyze 749,491 ≥20-year-old Taiwanese with physical/sensory disabilities in 2002–2008. The incidence of depression was analyzed by univariate Poisson regression. Risk factors of depression were followed up through 2014 and examined with a Cox proportional hazards model.

**Results:**

Among the study subjects, the incidence of depression was 6.29 per 1000 person-years, with 1.83 per 1000 person-years corresponding to major depression. The subjects’ depression risk was affected by disability type, disability severity, gender, age, education, marital status, aboriginal status, monthly salary, residence urbanization level, and Charlson comorbidity index (CCI). Subjects with rare diseases, mild disability, female gender, age 35–44 years, a high school education level, divorced/widowed status, non-aboriginal status, a NT$22,801–28,800 monthly salary, a highly urbanized residence area, or a CCI≥3 were at higher risk for depression.

**Conclusions and implications:**

Adults with physical/sensory disabilities have a 3.7-fold higher incidence of depression than the general population. Social services departments and family members should take extra measures toward preventing and treating depression in this subpopulation.

## Introduction

An estimated 350 million of the world’s population live with depression, and on average about 1 in 20 individuals surveyed in 2012 had an episode of depression in the previous year [1]. Depression imposes a considerable healthcare financial burden and presents a serious challenge to public health. Taiwan spends an average of US$116.6 million a year (about 1.2% of the total national healthcare expenditure) on treatment of depression [2]. Previous studies have shown that factors such as gender, family history of depression [3], health status, social support [4], and economic status [5] can affect the risk of depression.

Depression and physical illness or disability are correlated. Depression was positively associated with physical illness across age and gender [6]. Physical disability is a potential risk factor for depression, and depressive symptoms and physical disability combined can have a negative impact on physical and psychological health [7]. Compared with healthy individuals, individuals with physical disability reported more pain, depression, and anxiety and had a lower quality of life [8]. The prevalence of depression in adults with disabilities has been estimated at 24.9% [9] to 41% [10], higher than the 22.8% [9] to 27.5% [5] observed in normal adults. In a United States study, characteristics that increased the risk of severe depression in women with physical disabilities were shown to include younger age, greater problems with pain, more limited mobility, and less satisfaction with one’s social network [11].

Over 1 billion people worldwide have disabilities [12]. In Taiwan, there are 1.142 million people with disabilities as of 2014, representing 4.9% of the total population [13]. Although individuals with physical and sensory disabilities are at increased risk for depression, depression in these individuals has not been extensively studied in most countries. The present study examined the incidence, risk, and associated factors of depression in adults with physical and sensory disabilities, information that can potentially aid the prevention and treatment of depression in individuals with physical and sensory disabilities.

## Materials and methods

### Data sources and study subjects

The study subjects consisted of ≥ 20-year-old Taiwanese adults from year 2002 to 2008 who had a physical or sensory disability (including physical disability, visual impairment, hearing impairment, sound or speech impairment, multiple disabilities, major organ malfunction, facial injury, balance impairment, refractory epilepsy, and rare diseases). There were a total of 1,023,438 Taiwanese with disabilities. After excluding those whose disabilities were non-physical (including mental disabilities, senile dementia, autism, chromosome abnormalities, metabolic disorders, congenital defects, chronic psychosis, and chronic unconsciousness), 208,969; those in a vegetative state, 8,032; those aged <20 years, 27,461; those with missing values for gender and residence urbanization level, 231; and followed up through 2014 those who developed depression before acquiring their physical or sensory disability, 29,254, a total of 749,491 individuals remained who served as the subjects of this study. Data were obtained from two Taiwan national databases, including the Ministry of the Interior’s database of physically and mentally disabled persons and the NHI Research Database. The Institutional Review Board of Buddhist Dalin Tzu Chi Hospital approved this study (IRB No.B10501025).

### Description of variables

The independent variables examined included disability categories (type of disability and severity of disability), demographic characteristics (gender, age, education level, marital status, and aboriginal status), economic status (monthly salary), environmental factors (urbanization level of the area of residence), and health status (Charlson comorbidity index [CCI]). The dependent variable was whether the subject developed depression.

Further variable definitions are as follows: (a) The determination of depression (either major or minor depression) was based on at least three outpatient visits or one hospitalization in which the primary or secondary ICD-9-CM diagnosis code was 296.2, 296.3, 300.4, or 311 and in which an antidepressant (with the ATC code N06A) was prescribed. The first day of medical care that met the above criteria was taken to be the start date of depression. (b) The determination of major depression was based on at least three outpatient visits or one hospitalization in which the primary or secondary ICD-9-CM diagnosis code was 296.2 or 296.3. (c) Types of disability included 10 categories: physical disability, visual impairment, hearing impairment, sound or speech impairment, multiple disabilities, major organ malfunction, facial injury, balance impairment, refractory epilepsy, and rare diseases. (d) The severity of disability was categorized as mild, moderate, severe, or very severe. (e) The urbanization level of the area of residence was based on the urbanization level at the location of the subject’s health insurance enrollment and may be one of seven levels. Level 1 represents the most highly urbanized areas, whereas level 7 areas are the least urbanized. (f) The monthly salary level was specified by five categories: ≤NT$17,280, NT$17,281–22,800, NT$22,801–28,800, NT$28,801–36,300, and ≥NT$36,301. (g) The CCI as described by Deyo, Cherkin, and Ciol [14], used as a measure of comorbidity, was calculated by converting the study subject’s primary and secondary ICD-9-CM diagnosis codes—excluding those for depression—into weighted numerical scores and then summing them to obtain a final score, categorized as 0, 1, 2, or ≥3.

### Statistical analysis

In this retrospective cohort study, SAS statistical analysis software, version 9.3 (SAS Institute, Cary, NC, U.S.A.), was used to perform various statistical analyses.

Descriptive statistics were used to analyze the number of subjects, the number of new depression cases, and the incidence density rate (IDR = number of new cases/person-years*1000) with respect to the subjects’ disability categories (type of disability and severity of disability), demographic characteristics (gender, age, education level, marital status, and aboriginal status), economic status (monthly salary), environmental factors (urbanization level of the residence area), and health status (CCI). Univariate Poisson regression was performed to analyze the incidence rates of depression compared to the reference group in the subjects. The Cox proportional hazards model with stepwise approach was used to examine factors associated with the subjects’ risk of depression or major depression. The hazard ratio (HR) would be the relative risk of incidence of depression or major depression for the relevant variables in the models.

## Results

### Basic characteristics of adults with physical and sensory disabilities

As shown in Table 1, the majority of adults with physical and sensory disabilities had a physical disability (*n* = 391,848, 52.3%), and the severity of disability was most commonly categorized as mild (*n* = 279,964, 37.4%). The gender ratio was about 59% male to 41% female. The largest numbers of adults with physical and sensory disabilities were ≥65 years of age (*n* = 320,977, 42.8%), had a monthly salary in the NT$17,281–22,800 range (*n* = 328,735, 43.9%), resided in urbanization level 2 areas (moderately urbanized cities) (*n* = 202,779, 27.1%), and had a CCI score of 0 (*n* = 298,582, 39.8%).

**Table 1:** Incidence of depression in adults with physical and sensory disabilities, per 1000 person-years.

		Depression					Major depression				Dysthymic disorder			
Variable		*N*	New cases	Mean person- years	IDR^a^	*p*^b^	New cases	Mean person- years	IDR^a^	*p*^b^	New cases	Mean person- years	IDR^a^	*p*^b^
Total		749,491	30,489	6.46	6.29	-	9,038	6.59	1.83	-	21,451	6.52	4.39	-
Type of disability														
	Physical disability (ref.)	391,848	16,635	6.92	6.13	-	4,908	7.05	1.78	-	11,727	6.98	4.29	-
	Visual impairment	48,502	2,018	7.01	5.94	0.161	586	7.14	1.69	0.277	1,432	7.07	4.18	0.358
	Hearing impairment	91,129	3,605	6.81	5.81	0.003*	989	6.94	1.56	<0.001*	2,616	6.86	4.18	0.266
	Sound or speech impairment	11,633	395	6.65	5.10	<0.001*	130	6.75	1.66	0.423	265	6.70	3.40	<0.001*
	Multiple disabilities	78,617	2,640	5.57	6.03	0.396	724	5.67	1.62	0.025*	1,916	5.61	4.34	0.597
	Major organ malfunction	120,341	4,811	5.10	7.84	<0.001*	1,584	5.21	2.53	<0.001*	3,227	5.15	5.20	<0.001*
	Facial injury	3,533	154	6.34	6.87	0.161	56	6.46	2.45	0.016*	98	6.42	4.32	0.938
	Balance impairment	1,905	101	4.94	10.74	<0.001*	24	5.09	2.47	0.105	77	5.00	8.09	<0.001*
	Refractory epilepsy	1,639	107	6.13	10.65	<0.001*	27	6.34	2.60	<0.049*	80	6.22	7.85	<0.001*
	Rare diseases	344	23	5.20	12.86	<0.001*	10	5.31	5.48	<0.001*	13	5.30	7.13	0.067
Severity of disability					000D									
	Mild (ref.)	279,964	11,854	6.94	6.10	-	3,540	7.07	1.79	-	8,314	7.00	4.24	-
	Moderate	226,623	9,904	6.82	6.41	<0.001*	2,921	6.96	1.85	0.154	6,983	6.88	4.48	<0.001*
	Severe	138,985	5,209	5.82	6.44	<0.001*	1,483	5.93	1.80	0.854	3,726	5.87	4.57	<0.001*
	Very severe	103,919	3,522	5.27	6.43	<0.005*	1,094	5.36	1.96	0.007*	2,428	5.31	4.40	0.112
Gender														
	Male (ref.)	445,385	16,013	6.49	5.54	-	4,560	6.60	1.55	-	11,453	6.54	3.93	-
	Female	304,106	14,476	6.42	7.41	<0.001*	4,478	6.57	2.24	<0.001*	9,998	6.49	5.06	<0.001*
Age (years)														
	20–34 (ref.)	74,845	2,908	8.17	4.75	-	1,009	8.30	1.62	-	1,899	8.24	3.08	-
	35–44	103,273	4,836	8.18	5.72	<0.001*	1,660	8.33	1.93	<0.001*	3,176	8.26	3.72	<0.001*
	45–54	125,278	5,832	7.24	6.43	<0.001*	1,820	7.39	1.97	<0.001*	4,012	7.31	4.38	<0.001*
	55–64	125,118	5,911	6.57	7.19	<0.001*	1,671	6.72	1.99	<0.001*	4,240	6.63	5.11	<0.001*
	≥65	320,977	11,002	5.17	6.63	<0.001*	2,878	5.27	1.70	0.209	8,124	5.21	4.86	<0.001*
Education level														
	Elementary or illiterate (ref.)	385,435	15,026	6.27	6.22	-	4,204	6.38	1.71	-	10,822	6.32	4.45	-
	Junior high school	98,288	4,455	7.29	6.22	0.994	1,382	7.42	1.89	<0.001*	3,073	7.35	4.25	0.029*
	Senior or vocational high school	98,783	4,605	7.23	6.45	0.032*	1,482	7.37	2.03	<0.001*	3,123	7.30	4.33	0.193
	Junior college or university or above	49,973	2,100	6.95	6.05	0.228	642	7.08	1.81	0.156	1,458	7.01	4.16	0.018*
	Unknown	117,012	4,303	5.58	6.59	<0.001*	1,328	5.69	1.99	<0.001*	2,975	5.63	4.51	0.469
Marital status														
	Single (ref.)	126,192	4,878	7.29	5.30	-	1,609	7.41	1.72	-	3,269	7.36	3.52	-
	Married	404,875	16,363	6.16	6.56	<0.001*	4,695	6.28	1.85	0.015*	11,668	6.22	4.64	<0.001*
	Divorced or widowed	28,370	1,645	7.19	8.06	<0.001*	558	7.37	2.67	<0.001*	1,087	7.30	5.25	<0.001*
	Unknown	190,054	7,603	6.45	6.21	<0.001*	2,176	6.58	1.74	0.720	5,427	6.50	4.39	<0.001*
Aboriginal														
	No	733,849	29,963	6.45	6.33	-	8,852	6.57	1.84	-	21,111	6.50	4.42	-
	Yes	15,642	526	7.27	4.62	<0.001*	186	7.37	1.61	0.083	340	7.33	2.96	<0.001*
Monthly salary (NT$)														
	≤17,280^c^ (ref.)	88,297	3,253	7.51	4.91	-	1,052	7.62	1.56	-	2,201	7.56	3.30	-
	17,281–22,800	328,735	12,928	6.25	6.30	<0.001*	3,703	6.37	1.77	<0.001*	9,225	6.30	4.45	<0.001*
	22,801–28,800	142,246	6,011	5.64	7.50	<0.001*	1,884	5.75	2.30	<0.001*	4,127	5.69	5.09	<0.001*
	28,801–36,300	86,546	3,965	7.51	6.10	<0.001*	1,131	7.66	1.70	0.044*	2,834	7.58	4.32	<0.001*
	≥36,301	103,667	4,332	6.53	6.40	<0.001*	1,268	6.66	1.84	<0.001*	3,064	6.59	4.48	<0.001*
Urbanization of residence area^d^														
	Level 1 (ref.)	160,383	6,681	6.14	6.79	-	2,013	6.26	2.00	-	4,668	6.20	4.70	-
	Level 2	202,779	8,905	6.58	6.67	0.280	2,771	6.72	2.03	0.610	6,134	6.65	4.55	0.101
	Level 3	114,371	4,587	6.78	5.92	<0.001*	1,282	6.90	1.62	<0.001*	3,305	6.83	4.23	<0.001*
	Level 4	141,357	5,644	6.54	6.10	<0.001*	1,650	6.67	1.75	<0.001*	3,994	6.60	4.28	<0.001*
	Level 5	27,985	1,004	6.51	5.51	<0.001*	289	6.62	1.56	<0.001*	715	6.56	3.90	<0.001*
	Level 6	53,388	1,914	6.26	5.73	<0.001*	559	6.36	1.65	<0.001*	1,355	6.31	4.02	<0.001*
	Level 7	49,228	1,754	6.29	5.66	<0.001*	474	6.40	1.50	<0.001*	1,280	6.34	4.10	<0.001*
CCI score														
	0 (ref.)	298,582	9,264	8.23	3.77	-	2,834	8.33	1.14	-	6,430	8.28	2.60	-
	1	126,885	6,036	6.76	7.04	<0.001*	1,748	6.91	1.99	<0.001*	4,288	6.83	4.95	<0.001*
	2	110,502	5,549	5.91	8.50	<0.001*	1,605	6.06	2.40	<0.001*	3,944	5.98	5.97	<0.001*
	≥3	213,522	9,640	4.11	10.98	<0.001*	2,851	4.24	3.15	<0.001*	6,789	4.17	7.63	<0.001*

^a^Incidence density rate (IDR) = number of new cases/person-years*1000.

^b^Univariate Poisson regression analysis.

^c^The category included low-income households.

^d^Level 1corresponds to the most urbanized areas.

*p < 0.05.

N = number = 749,491

HR = hazard ratio

CI = confidence interval

CCI = Charlson comorbidity index

IDR = the incidence density rate

### Incidence of depression (major and minor depression) in adults with physical and sensory disabilities

In Table 1, univariate Poisson regression analysis showed that the incidence rate of depression in adults with physical and sensory disabilities was 6.29 per 1000 person-years. The incidence varied significantly with factors including type of disability, severity of disability, gender, age, education level, marital status, aboriginal status, monthly salary, urbanization level of the residence area, and CCI.

With respect to the type of disability, major organ malfunction, balance impairment, refractory epilepsy, and rare diseases were associated with an increased incidence of depression (7.84–12.86 per 1000 person-years) relative to physical disability (6.13 per 1000 person-years), with the incidence being highest for rare diseases (12.86 per 1000 person-years) (*p* < 0.05). With respect to the severity of disability, those with moderate, severe, and very severe disability had a higher incidence of depression (6.41–6.44 per 1000 person-years) than those with mild disability (6.10 per 1000 person-years), and the incidence was highest among those with severe disability (6.44 per 1000 person-years) (*p* < 0.05).

By gender, the incidence of depression was higher in women than men with physical and sensory disabilities (7.41 vs. 5.54 per 1000 person-years) (*p* < 0.05). By age, all ≥35-year groups had a greater incidence of depression (5.72–7.19 per 1000 person-years) than the 20–34-year group (4.75 per 1000 person-years), with the 55–64-year group having the highest incidence (7.19 per 1000 person-years) (*p* < 0.05). As for marital status, being married, divorced or widowed, or of unknown marital status was associated with a higher incidence of depression (6.21–8.06 per 1000 person-years) than being single (5.30 per 1000 person-years), with the highest incidence being observed for being divorced or widowed (8.06 per 1000 person-years) (*p* < 0.05).

With respect to aboriginal status, aboriginals with physical and sensory disabilities had a lower incidence of depression than their non-aboriginal counterparts (4.62 vs. 6.33 per 1000 person-years) (*p* < 0.05). In terms of monthly salary, all ≥NT$17,281 categories were associated with an increased incidence of depression (6.10–7.50 per 1000 person-years) relative to the ≤NT$17,280 category (4.91 per 1000 person-years) (*p* < 0.05). In terms of the urbanization level of the residence area, those residing in level 3 to level 7 areas had a lower incidence of depression (5.51–6.10 per 1000 person-years) than those residing in urbanization level 1 areas (6.79 per 1000 person-years) (*p* < 0.05). As for comorbidity, all of the CCI≥1 categories were correlated with a higher incidence of depression (7.04–10.98 per 1000 person-years) than the CCI category of 0 (3.77 per 1000 person-years). Moreover, the incidence of depression increased with increasing CCI score and was highest for CCI scores ≥3 (10.98 per 1000 person-years) (*p* < 0.05).

### Incidence of major depression in adults with physical and sensory disabilities

The incidence of major depression in adults with physical and sensory disabilities was 1.83 per 1000 person-years (Table 1). Among the types of disability, major organ malfunction, facial injury, refractory epilepsy, and rare diseases were associated with an increased incidence of major depression (2.45–5.48 per 1000 person-years) relative to physical disability (1.78 per 1000 person-years), with the incidence being highest for rare diseases (5.48 per 1000 person-years) (*p* < 0.05). In terms of the severity of disability, those with very severe disability had a greater incidence of major depression than those with mild disability (1.96 vs. 1.79 per 1000 person-years) (*p* < 0.05).

By gender, the incidence of major depression was higher in women than men with physical and sensory disabilities (2.24 vs. 1.55 per 1000 person-years) (*p* < 0.05). Except for the ≥65-year age group whose result was not statistically significant, the incidence of major depression increased with increasing age, with the 55–64-year group having the highest incidence (1.99 per 1000 person-years) (*p* < 0.05). With respect to marital status, being married or divorced or widowed was correlated with an increased incidence of major depression (1.85–2.67 per 1000 person-years) relative to being single (1.72 per 1000 person-years), with the highest incidence being observed for being divorced or widowed (2.67 per 1000 person-years) (*p* < 0.05).

With respect to monthly salary, all ≥NT$17,281 categories were associated with a higher incidence of major depression (1.70–2.30 per 1000 person-years) than the ≤NT$17,280 category (1.56 per 1000 person-years) (*p* < 0.05). In terms of the urbanization level of the residence area, those residing in level 3 to level 7 areas had a lower incidence of major depression (1.50–1.75 per 1000 person-years) than those residing in urbanization level 1 areas (2.00 per 1000 person-years) (*p* < 0.05). As for comorbidity, the incidence of major depression increased with increasing CCI score, with the highest incidence being for CCI scores ≥3 (3.15 per 1000 person-years) (*p* < 0.05).

### Risk of depression and associated factors in adults with physical and sensory disabilities

In Table 2, as revealed by Cox proportional hazards modeling, factors that significantly influenced the risk of depression in adults with physical and sensory disabilities (*p* < 0.05) included type of disability, severity of disability, gender, age, education level, marital status, aboriginal status, monthly salary, urbanization level of the residence area, and CCI. With respect to the type of disability, balance impairment, refractory epilepsy, and rare diseases were correlated with a 1.30–1.90-fold increased risk of depression relative to physical disability (*p* < 0.05), with the risk being highest for rare diseases (HR = 1.90, 95% CI = 1.26–2.86) (*p* < 0.05). With respect to the severity of disability, those with very severe disability had a lower risk of depression than those with mild disability, the reference group (HR = 0.72, 95% CI = 0.69–0.76) (*p* < 0.05).

**Table 2:** Risk of depression and associated factors in adults with physical and sensory disabilities.

		Depression				Major depression			
Variable		HR	95% CI		*p*	HR	95% CI		*p*
Type of disability									
	Physical disability (ref.)	1.00	-	-	-	1.00	-	-	-
	Visual impairment	1.02	0.97	1.07	0.385	1.13	1.04	1.24	0.005*
	Hearing impairment	0.99	0.96	1.03	0.706	1.06	0.99	1.14	0.086
	Sound or speech impairment	0.78	0.70	0.86	<0.001*	0.85	0.71	1.01	0.060
	Multiple disabilities	0.99	0.95	1.04	0.744	0.86	0.79	0.93	<0.001*
	Major organ malfunction	0.95	0.92	0.99	0.019*	1.02	0.95	1.10	0.517
	Facial injury	0.97	0.83	1.14	0.694	1.07	0.82	1.39	0.636
	Balance impairment	1.30	1.07	1.58	0.009*	1.03	0.69	1.55	0.870
	Refractory epilepsy	1.79	1.48	2.16	<0.001*	1.24	0.85	1.81	0.272
	Rare diseases	1.90	1.26	2.86	0.002*	2.28	1.22	4.23	0.010*
Severity of disability									
	Mild (ref.)	1.00	-	-	-	1.00	-	-	-
	Moderate	1.00	0.97	1.02	0.754	0.99	0.94	1.04	0.643
	Severe	0.97	0.94	1.01	0.138	0.83	0.78	0.88	<0.001*
	Very severe	0.72	0.69	0.76	<0.001*	0.67	0.62	0.73	<0.001*
Gender									
	Male (ref.)	1.00	-	-	-	1.00	-	-	-
	Female	1.34	1.31	1.37	<0.001*	1.57	1.50	1.64	<0.001*
Age (years)									
	20–34 (ref.)	1.00	-	-	-	1.00	-	-	-
	35–44	1.07	1.02	1.12	0.004*	1.05	0.97	1.14	0.242
	45–54	1.03	0.98	1.08	0.214	0.90	0.83	0.97	0.010*
	55–64	1.02	0.97	1.07	0.500	0.78	0.71	0.85	<0.001*
	≥65	0.87	0.83	0.91	<0.001*	0.49	0.45	0.53	<0.001*
Education level									
	Elementary school or illiterate (ref.)	1.00	-	-	-	1.00	-	-	-
	Junior high school	1.16	1.12	1.20	<0.001*	1.15	1.08	1.23	<0.001*
	Senior or vocational high school	1.19	1.15	1.24	<0.001*	1.23	1.15	1.31	<0.001*
	Junior college or university or above	1.09	1.04	1.15	<0.001*	1.10	1.00	1.20	0.041*
	Unknown	1.07	1.03	1.11	<0.001*	1.02	0.96	1.09	0.502
Marital status									
	Single (ref.)	1.00	-	-	-	1.00	-	-	-
	Married	1.08	1.05	1.12	<0.001*	0.97	0.91	1.03	0.271
	Divorced or widowed	1.29	1.22	1.37	<0.001*	1.54	1.40	1.70	<0.001*
	Unknown	1.04	1.00	1.08	0.040*	0.90	0.84	0.96	0.002*
Aboriginal									
	No (ref.)	1.00	-	-	-				
	Yes	0.78	0.72	0.85	<0.001*				
Monthly salary (NT$)									
	≤17,280 (ref.)	1.00	-	-	-	1.00	-	-	-
	17,281–22,800	1.02	0.98	1.06	0.385	0.89	0.83	0.96	0.002*
	22,801–28,800	1.19	1.14	1.24	<0.001*	1.01	0.94	1.09	0.719
	28,801–36,300	1.03	0.98	1.08	0.245	1.00	0.92	1.09	0.935
	≥36,301	1.00	0.96	1.05	0.956	0.91	0.84	0.99	0.022*
Urbanization of residence area ^d^									
	Level 1 (ref.)	1.00	-	-	-	1.00	-	-	-
	Level 2	0.98	0.95	1.01	0.262	1.05	0.99	1.11	0.144
	Level 3	0.92	0.89	0.96	<0.001*	0.89	0.83	0.96	0.002*
	Level 4	0.93	0.90	0.97	<0.001*	0.98	0.91	1.05	0.536
	Level 5	0.86	0.80	0.92	<0.001*	0.91	0.80	1.03	0.131
	Level 6	0.88	0.84	0.93	<0.001*	0.90	0.82	0.99	0.036*
	Level 7	0.87	0.83	0.92	<0.001*	0.81	0.73	0.90	<0.001*
CCI score									
	0 (ref.)	1.00	-	-	-	1.00	-	-	-
	1	1.83	1.77	1.89	<0.001*	1.72	1.62	1.83	<0.001*
	2	2.29	2.21	2.38	<0.001*	2.00	1.88	2.14	<0.001*
	≥3	2.93	2.84	3.03	<0.001*	2.08	1.96	2.21	<0.001*

^d^Level 1 corresponds to the most urbanized areas.

*p < 0.05.

N = number = 749,491

HR = hazard ratio

CI = confidence interval

CCI = Charlson comorbidity index

By gender, the risk of depression was higher in women than men with physical and sensory disabilities (HR = 1.34, 95% CI = 1.31–1.37) (*p* < 0.05). Relative to the 20–34-year age group, depression risk was increased in the 35–44-year group (HR = 1.07, 95% CI = 1.02–1.12) (*p* < 0.05) and decreased in the ≥65-year group (HR = 0.87, 95% CI = 0.83–0.91) (*p <* 0.05). In terms of education, the education levels of junior high school, senior or vocational high school, junior college or university or above, and unknown were associated with a higher risk of depression (HR = 1.07–1.19) than the education level of illiterate or elementary school, with the risk being highest for senior or vocational high school education (HR = 1.19, 95% CI = 1.15–1.24) (*p* < 0.05). As for marital status, those who were married, divorced or widowed, or of unknown marital status were at greater risk of depression (HR = 1.04–1.29) than those who were single, with the risk being highest in those who were divorced or widowed (HR = 1.29, 95% CI = 1.22–1.37) (*p* < 0.05).

With respect to aboriginal status, aboriginals with physical and sensory disabilities had a lower risk of depression (HR = 0.78, 95% CI = 0.72–0.85) than their non-aboriginal counterparts (*p* < 0.05). In terms of monthly salary, the NT$22,801–28,800 category was associated with a higher risk of depression (HR = 1.19, 95% CI = 1.14–1.24) than the ≤NT$17,280 category (*p* < 0.05). As for the urbanization level of the residence area, the risk of depression was lower for those residing in level 3 to level 7 areas (HR = 0.86–0.93) than for those residing in urbanization level 1 areas (*p* < 0.05). Compared with the CCI category of 0, all of the CCI≥1 categories were correlated with an increased risk of depression (HR = 1.83–2.93); the risk grew as the CCI score increased, with the highest risk being observed for CCI scores ≥3 (HR = 2.93, 95% CI = 2.84–3.03) (*p* < 0.05).

### Risk of major depression and associated factors in adults with physical and sensory disabilities

Factors that affected the risk of major depression in adults with physical and sensory disabilities included type of disability, severity of disability, gender, age, education level, marital status, monthly salary, urbanization level of the residence area, and CCI (Table 2). Among the types of disability, visual impairment and rare diseases were associated with a 1.13–2.28-fold increased risk of major depression relative to physical disability (*p* < 0.05), with the risk being highest for rare diseases (HR = 2.28, 95% CI = 1.22–4.23) (*p* < 0.05). In terms of the severity of disability, severe and very severe disability were correlated with lower risk of major depression (HR = 0.67–0.83) than was mild disability, the reference category (*p* < 0.05).

By gender, the risk of major depression was higher in women than men with physical and sensory disabilities (HR = 1.57, 95% CI = 1.50–1.64) (*p* < 0.05). By age, the 45-54-year, 55–64-year and ≥65-year groups had a lower risk of major depression (HR = 0.49–0.90) than the 20–34-year group (*p* < 0.05), with the ≥65-year group having the lowest risk (HR = 0.49, 95% CI = 0.45–0.53) (*p* < 0.05). In terms of education, the education levels of junior high school, senor or vocational high school, and junior college or university or above were associated with a higher risk of major depression (HR = 1.10–1.23) than the education level of illiterate or elementary school, with the highest risk being observed at the senior or vocational high school level (HR = 1.23, 95% CI = 1.15–1.31) (*p* < 0.05).

In terms of marital status, those who were divorced or widowed were at higher risk of major depression (HR = 1.54, 95% CI = 1.40–1.70) than those who were single (*p* < 0.05). As for monthly salary, the NT$17,281–22,800 and ≥36,301 category had a lower risk of major depression (HR = 0.89–0.91) than the ≤NT$17,280 category (*p* < 0.05). With respect to the urbanization level of the residence area, the risk of major depression was lower for residing in level 3, level 6 and level 7 areas (HR = 0.81–0.89) than for residing in level 1 areas (*p* < 0.05). As the CCI score increased, the risk of major depression also increased, and reached the highest level for CCI scores≥3 (HR = 2.08, 95% CI = 1.96–2.21) (*p* < 0.05).

## Discussion

As shown by our results, the incidence of depression in adults with physical and sensory disabilities was 6.29 per 1000 person-years, which is 3.7-fold higher than the incidence of depression previously reported for the general population (1.70 per 1000 person-years) [15].

Past studies have examined whether depression is related to the severity of disability. Barry and colleagues [16] found that among community-living elderly persons, the greater the severity of disability, the greater the likelihood of subsequent depression. In a study of adults in England by Meltzer et al. [17], limitations in activities of daily living (ADL) and in instrumental activities of daily living (IADL) were significantly associated with depression, with a seeming cumulative effect. However, our present analysis showed that among adults with physical and sensory disabilities, those whose disability was very severe had a lower risk of depression with those with mild disability. Because individuals with very severe disability rely on the assistance of family members or responsible institutions to access healthcare services, it is possible that depression in such individuals is more frequently overlooked and thus underestimated in national healthcare databases.

Concerning gender differences, it has been shown that women are more likely than men to have depression [18] and that depression is a significant problem in women with disabilities [19–21]. Many factors that are common in the lives of women with physical disabilities, including socioeconomic disadvantage, functional limitations, pain and other chronic health conditions, poor diet, physical inactivity, low self-esteem, and chronic stress, have been linked to higher rates of depression [22]. In addition, women may be more vulnerable to inflammation-induced mood and behavior changes than men [23]. Our results are consistent with the above studies.

Of the marital status categories we examined, divorced or widowed women with physical and sensory disabilities had the highest risk of depression (Table 2). Widowhood is well recognized as a stressful life experience and may be a trigger for depression [24]. The loss of a spouse was predictive of a higher incidence of depressive symptoms [25]. Compared with a still-married control group, widows and widowers scored significantly higher on loneliness, sadness, depressed mood, and appetite loss and significantly lower on happiness and enjoyment of life [26] and had worse depressive symptoms [27]. Another recent study concluded that spousal bereavement mainly affects loneliness, which in turn activates other depressive symptoms [26]. Our finding on the effect of marital status on depression in women with physical and sensory disabilities is in agreement with these previous research findings.

With respect to age, Kessler et al. [28] showed that depression affects all age groups, but especially the middle aged. Arango-Lasprilla and colleagues [29] found that among individuals with spinal cord injury, those aged 35–55 years had the highest odds of depression. Among Taiwanese adults, age ≤40years was associated with an increased risk of depression [30]. In the present study of adults with physical and sensory disabilities, the age group at greatest risk for depression was 35–44 years (Table 2). Our result is consistent with a conclusion from the other studies that a loss of employment or workdays due to injury and the resulting impact on family life are related to the increased depression risk of this age group.

Regarding the effect of the urbanization level of the residence area, we found that adults with physical and sensory disabilities living in level 3, level 6 and level 7 areas were at lower risk of depression than those living in the highly urbanized level 1 areas (Table 2). Aspects of rural living such as an idyllic physical environment, residential stability, close social ties, and stable social networks are considered factors that may reduce the risk of depression. Previous studies conducted in the United States [31], in India [32], in Canada [33], and in Taiwan [30] showed that adults living in rural areas had a lower risk of depression, in agreement with our finding of a lower depression risk for residence in less urbanized areas.

On the association between CCI score and the risk of depression, Harpole et al. [34] found that patients with more chronic diseases had higher depression severity. Consistently, our results (Table 2) show that the risk of depression in individuals with physical and sensory disabilities was exacerbated by increasing CCI scores.

### Study limitations

The main source of data for this study was Taiwan’s National Health Insurance Database; as a result, insurance premium–based monthly salaries rather than individuals’ monthly incomes were used in our analysis. Also, as individuals with more severe physical or sensory disabilities are often less able to notice the effects of their own depression and are thus less likely to request medical help for it, such cases tend to be overlooked by government social services departments, institutions for the disabled, and family members and not accurately reflected in the available data, thereby limiting our analysis. The discrepancy between our results and those of the above-cited authors is primarily due to the fact that these authors analyzed questionnaire responses from participants, whereas we analyzed healthcare outcomes compiled in national databases. This difference in study results can be a focus of further research.

## Conclusions

In summary, we found that the incidence of depression in adults with physical and sensory disabilities was 3.7 times that in the general population. Factors associated with an increased risk of depression in adults with physical and sensory disabilities included rare diseases, mild disability, female gender, age 35–44 years, a senior or vocational high school education level, being divorced or widowed, being non-aboriginal, a monthly salary of NT$22,801–28,800, residence in urbanization level 1 areas, and a CCI score ≥3. Given the high incidence of depression and the associated risk characteristics in individuals with physical and sensory disabilities, we suggest that government social services departments and family members focus more attention on detecting and securing medical care for depression in these individuals and implement extra measures to improve the prevention and treatment of depression for these individuals.

## Abbreviations

HR: hazard ratio
CI: confidence interval
CCI: Charlson comorbidity index
ICD-9-CM: International Classification of Diseases, Ninth Revision, Clinical Modification
IDR: the incidence density rate
ADL: activities of daily living
IADL: instrumental activities of daily living
ref.: reference group
